# Change in weight and BMI associated with switching to bictegravir/emtricitabine/tenofovir alafenamide versus a dolutegravir-based regimen among virologically suppressed adults living with HIV through 144 weeks

**DOI:** 10.1097/MD.0000000000041728

**Published:** 2025-03-07

**Authors:** Charlotte-Paige Rolle, Jeffrey Garrett, Jamie Castano, Vu Nguyen, Kiran Patel, Federico Hinestrosa, Edwin DeJesus

**Affiliations:** a Orlando Immunology Center, Orlando, FL; b Department of Global Health, Emory University Rollins School of Public Health, Atlanta, GA; c Gilead Sciences, Foster City, CA; d University of Central Florida College of Medicine, Orlando, FL.

**Keywords:** bictegravir, cardiometabolic, dolutegravir, INSTIs, treatment-experienced, virologically suppressed, weight gain

## Abstract

Increased weight has been observed among treatment-naïve-and-experienced people living with HIV initiating bictegravir (BIC) and dolutegravir (DTG). Here, we report changes in weight and body mass index (BMI) following switch to a BIC versus DTG-based regimen (DBR) through 144 weeks. This observational study collected demographics, clinical characteristics, weight, and BMI from virologically suppressed adults switched to BIC/emtricitabine/tenofovir alafenamide (TAF), emtricitabine/TAF plus DTG, DTG/abacavir/lamivudine, DTG/rilpivirine (RPV), and DTG/lamivudine 2 years prior to switch through 144 weeks post-switch. Linear spline models were fit to estimate and compare the trajectories of weight and BMI changes observed pre-and-post-switch. Adjusted piecewise linear mixed-effects models were fit to examine factors associated with weight and BMI change pre-and-post-switch. At week 144, switching to BIC/emtricitabine/TAF versus a DBR were both associated with lower annualized weight gain post-switch (‐0.88 kg/year vs −0.39 kg/year respectively, *P* = .15). DTG plus emtricitabine/TAF switches had the highest annualized weight gain (0.68 kg/year, 95% confidence interval: −0.32, 1.65) whereas, DTG/RPV switches had the lowest annualized weight gain (‐2.22 kg/year, 95% confidence interval: −3.69, −0.62) post-switch. DTG/RPV and BIC/emtricitabine/TAF switches were the only groups with significantly lower annualized weight gain post-switch at week 144. Baseline BMI < 18.5 kg/m^2^ was associated with the highest annualized weight gain post-switch, whereas switching from protease inhibitors and self-report of dieting were associated with the lowest annualized weight gain post-switch. At week 144, switching to a BIC versus DBR were both associated with lower annualized weight gain post-switch among a large and diverse cohort of treatment-experienced people living with HIV.

## 1. Introduction

Second-generation integrase strand transfer inhibitors (INSTIs) such as dolutegravir (DTG) and bictegravir (BIC) have been associated with increased weight and body mass index (BMI) among people living with HIV (PLWH) compared to other antiretrovirals (ARVs).^[1–12]^ However, few studies have compared weight gain associated with switching to DTG versus BIC among treatment-experienced PLWH and there is little data which links increased weight to worsening cardiometabolic outcomes in this population.

Pooled data from randomized clinical trials (RCTs) of 7316 virologically suppressed adults demonstrated greater weight gain among those switching ARVs with most weight gain occurring in the first 24 weeks following switch.^[13]^ Only younger age and lower baseline BMI was associated with ≥ 10% weight gain and switches from efavirenz (EFV) to rilpivirine (RPV) or elvitegravir/cobicistat (EVG/c) and tenofovir disoproxil fumarate (TDF) to tenofovir alafenamide (TAF) had the greatest risk of weight gain. There was no significant weight change with switch from DTG to BIC however greater weight gain was observed among those switching to BIC and continuing DTG compared to remaining on EVG/c. There was no significant impact on metabolic parameters and incidence of treatment-emergent diabetes among those who switched and had ≥ 10% weight gain.^[13]^

Results from the PASO-DOBLE RCT which evaluated weight changes among 553 virologically suppressed adults switching to BIC (B)/emtricitabine (F)/TAF versus DTG/lamivudine (3TC) from regimens containing ≥ 1 pill daily, cobicistat, EFV or TDF demonstrated greater mean weight change with B/F/TAF (1.81 kg, 95% confidence interval [CI]: 1.28–2.34) versus DTG/3TC (0.89 kg, 95% CI: 0.37–1.41) at week 48.^[14]^ Subgroup analyses demonstrated that risk of > 5% weight gain among those switched to DTG/3TC did not vary by pre-switch regimen whereas this risk was only increased among those switching to B/F/TAF from TDF and non-nucleoside reverse transcriptase inhibitors (NNRTIs).^[14]^

Other studies evaluating weight change with BIC and DTG include data from treatment-experienced PLWH switching to INSTIs identified in IQVIA’s Ambulatory Electronic Medical Records database.^[10]^ A cohort of 803 PLWH switching to INSTIs from NNRTIs and protease inhibitors (PIs) revealed that those who switched to INSTIs from an NNRTI had higher odds of ≥ 5 % weight gain compared to those remaining on an NNRTI. The risk of weight gain was greatest among switches to BIC followed by DTG and EVG. PLWH switching from a PI to INSTI did not have an increased risk of ≥ 5% weight gain compared to those remaining on PIs regardless of INSTI agent. Notably, 85% switching from NNRTIs switched from EFV and 91% switched from TDF. This may explain these findings given that EFV and TDF are thought to be weight-suppressive and their removal from ARV regimens has been associated with weight gain.^[15,16]^

Data from another real-world cohort demonstrated that among 236 switched to INSTIs, significant weight gain was observed with switch to all INSTI agents except for BIC.^[9]^ Discontinuation of INSTIs did not result in significant weight change except for those discontinuing raltegravir (RAL) who continued to gain weight post-switch. Notably, only 47/236 switched to BIC and only 14/236 switched to RAL hence this data may be limited by small sample sizes in those groups.

Other observational analyses evaluating weight change following switch to INSTIs did not include BIC and have demonstrated mixed results with some reporting significantly increased weight following switch, particularly to DTG^[3–6,17]^ and others reporting no significant weight change regardless of INSTI type.^[18–20]^

Few studies have evaluated the impact of weight changes on cardiometabolic parameters, and the data is also mixed. Analyses from the RESPOND cohort have linked increases in BMI following INSTI initiation to an increased risk of hypertension, hyperlipidemia, and cardiovascular disease among PLWH.^[21,22]^ Data from commercially and Medicaid-insured treatment-naïve PLWH demonstrated that INSTI use was associated with incident diabetes mellitus and hyperglycemia 6 months following initiation.^[23]^

In contrast, other reports have failed to demonstrate a consistent link between INSTI-associated weight gain and new-onset cardiometabolic disease. Among 495 virologically suppressed adults who gained weight following switch to an INSTI from an NNRTI containing regimen, no significant changes were observed in hemoglobin A1c (HbA1c).^[8]^ Data from another study of 349 PLWH did not reveal any significant difference in glucose levels, mean blood pressure and lipid parameters among those switching to an INSTI versus those remaining on non-INSTI regimens despite more weight gain in the INSTI switch group.^[24]^ Results from virologically suppressed women participating in the Women’s Interagency HIV study demonstrated increased weight and BMI following switch to INSTIs with small but significantly greater increases in HbA1c, systolic blood pressure, and diastolic blood pressure but no significant difference in the development of diabetes mellitus and hypertension between the INSTI and non-INSTI switch groups.^[17]^

Overall, the relationship between INSTIs and weight gain remains unclear, with little data on the impact of newer INSTIs. It is imperative to understand the differential effects of BIC and DTG on weight, BMI and cardiometabolic parameters as both are guideline-preferred ARV options for most PLWH and this may be an important differentiator for populations more prone to weight gain and metabolic disease. Here we report change in weight, BMI, and cardiometabolic parameters from a prospective longitudinal cohort of virologically suppressed adults switched to B/F/TAF versus a DTG-based regimen (DBR) through 144 weeks compared to 2 years prior to switch.

## 2. Methods

This is a prospective longitudinal study to compare pre-and-post-switch changes in weight, BMI, and cardiometabolic parameters among virologically suppressed adults switched to B/F/TAF versus a DBR at the Orlando Immunology Center (OIC) through 144 weeks. Eligible participants included all PLWH switched to B/F/TAF (fixed-dose combination (FDC)), DTG plus F/TAF, DTG/abacavir (ABC)/3TC (FDC), DTG/RPV (FDC) or DTG/3TC (FDC) as a complete regimen between February 7th, 2018, and July 31st, 2020. Other inclusion criteria included the availability of 2 consecutive baseline HIV-1 RNA values < 50 copies/mL (at least 3 months apart) in the year prior to switch, attendance at ≥ 4 clinic visits with corresponding weight and BMI values in the 2 years prior to switch and attendance at ≥ 2 clinic visits with corresponding weight and BMI values in the year following switch. PLWH were excluded if they were pregnant, had unstable thyroid disease (defined as an abnormal thyroid stimulating hormone value within 3 months of baseline), baseline Grade 3 or 4 laboratory abnormalities, and missing weight or BMI values in the 2 years prior to switch.

Demographics, lab values, clinical parameters and data on weight, BMI, and cardiometabolic factors were collected from the electronic medical record (EMR) 2 years prior to switch through 144 weeks post-switch. Weight and height are measured as a part of the vital signs assessment at every OIC clinic visit using the same scale which is calibrated bi-annually. BMI is calculated at every visit based on these measurements. Reasons for ARV switch were obtained from a templated “ARV switch” subjective, objective, assessment, and plan note used by providers to document decisions surrounding ARV switch. The following specific data were collected for this study: lipid panel, fasting glucose, HbA1c, tobacco use, referral to the OIC wellness clinic (the latter is focused on helping PLWH achieve healthy weight loss and is staffed by OIC clinicians with advanced training on nutrition, exercise counseling and management of weight loss pharmacotherapy), self-report of physical activity (if documented in the EMR), self-report of dieting (if documented in the EMR), psychiatric comorbidities (based on the presence of the following diagnoses documented in the medical history: depression, anxiety, bipolar disorder, attention deficit disorder, attention deficit hyperactivity disorder, post-traumatic stress disorder, schizophrenia, substance use disorder, obsessive compulsive disorder, and personality disorder), use of medications associated with weight loss (based on documented prescription of the following drugs during the study period regardless of dose or dose changes: orlistat, lorcaserin, buproprion/naltrexone, metformin, semaglutide, liraglutide, phentermine, and phentermine-topiramate), use of medications associated with weight gain (based on documented prescription of the following drugs during the study period regardless of dose or dose changes: antipsychotics, all antidepressants except for fluoxetine and buproprion, mood stabilizers, anticonvulsants, corticosteroids, sulfonylureas, meglitinides, thiazolidinediones, insulin, oral hormonal contraceptives, atenolol, propranolol, and metoprolol), new diagnosis of cardiometabolic disease (based on the addition of the following diagnoses to the medical history during the study period: hypertension, hyperlipidemia, obesity, diabetes mellitus, coronary artery disease, ischemic heart disease, cerebrovascular accident, transient ischemic attack, peripheral vascular disease, peripheral arterial disease, and metabolic dysfunction-associated steatotic liver disease (MASLD)) and changes in the use of medications to treat the latter conditions (based on new or discontinued prescription of a drug during the study period regardless of dose or dose changes and documentation that the drug was being used for one of the cardiometabolic diagnoses previously mentioned).

The primary endpoint was annualized change in weight and BMI among those switching to B/F/TAF versus a DBR through 144 weeks post-switch compared to 2 years pre-switch. Secondary endpoints included changes in cardiometabolic factors (incident cardiometabolic disease, use of medications to treat cardiometabolic disease, referral to the OIC wellness clinic and use of weight loss pharmacotherapy) among PLWH switching to B/F/TAF versus a DBR at week 144 and evaluation of demographic and clinical factors associated with changes in weight pre-and post-switch.

Descriptive statistics were used to summarize participant baseline demographic and clinical characteristics. Locally estimated scatterplot smoothing curves were used to plot weight and BMI by study regimen. Within-person weight and BMI changes were estimated pre-and-post-INSTI switch to allow participants to serve as their own controls for estimation of background and age-related weight gain. Linear spline models with a knot at the time of switch were fit to estimate and compare the trajectories of annualized weight and BMI changes observed pre-and-post-switch. Models were adjusted for age, sex, race, ethnicity, baseline BMI, baseline CD4^+^ T-cell count, nadir CD4^+^ T-cell count, duration of HIV infection, pre-switch nucleoside reverse transcriptase inhibitor (NRTI), smoking status, baseline psychiatric disease, use of medications associated with weight loss, use of medications associated with weight gain, referral to the OIC wellness clinic, self-report of physical activity, and self-report of dieting. Participants who stopped or switched the study regimen were censored at the time of discontinuation or switch. Participants in the DTG plus F/TAF group who changed the dose of their study regimen components remained in the study. The proportion of participants in each treatment group with new-onset cardiometabolic disease, starting or discontinuing medications for cardiometabolic disease, referred to the OIC wellness clinic and initiating weight loss pharmacotherapy through 144 weeks was compared using unadjusted chi-square tests. Piecewise linear mixed-effects models adjusting for age, sex, race, ethnicity, baseline BMI, baseline CD4^+^ T-cell count, pre-switch anchor drug, pre-switch NRTI, smoking status, baseline psychiatric disease, any use of medications associated with weight gain, any use of medications associated with weight loss, referral to the OIC wellness clinic, self-report of physical activity and self-report of dieting were fit to examine factors associated with weight, and BMI change pre-and-post-switch. *P* < .05 (two-sided) was considered statistically significant. All statistical analyses were conducted using RStudio, version 4.3.

The Sterling Institutional Review Board (IRB) determined that the study met IRB exemption criteria based on the observational nature of the analysis which utilized data collected as a part of routine clinical care (Sterling IRB ID 7930). Informed consent was not utilized for this observational cohort and was determined to not be required by the Sterling IRB.

## 3. Results

Between February 7th, 2018 to July 31st, 2020, 1973 PLWH were switched to B/F/TAF or DBRs however, 204 did not have 2 consecutive baseline HIV-1 RNA values < 50 copies/mL in the year prior to switch, 662 did not have weight/BMI data available in the 2 years prior to switch and 151 did not have at least 2 weight/BMI measurements in the year following switch leaving 956 eligible for study inclusion. Of 956, 673 switched to B/F/TAF, 148 switched to DTG plus F/TAF, 51 switched to DTG/ABC/3TC, 48 switched to DTG/RPV, and 36 switched to DTG/3TC. At baseline the median age (range) was 53 (21–83) years, 15% were cisgender women, 45% were non-White, median weight (range) was 85 (43.9–185.2) kg, and median BMI (range) was 27.9 (14.3–66.5) kg/m^2^ (Table 1). Among B/F/TAF switches, 401 (60%) switched from a dual NRTI + INSTI, 472 (70%) switched from TAF-based regimens, 298 (44%) switched from a regimen containing RAL or EVG/c and 103 (16%) switched from a DBR. The most commonly documented reason for switch was simplification. Among DBR switches, 147 (52%) switched from a dual NRTI + INSTI, 116 (41%) switched from TDF-based regimens, 50 (18%) switched from a regimen containing RAL or EVG/c and 91 (32%) switched from another DBR. The most commonly documented reason for switch was simplification (Table 1).

**Table 1:** Baseline demographic and clinical characteristics.

Characteristic	N = 956
Median age (range)	53 (21, 83)
Sex	
Male, n (%)	809 (85)
Female, n (%)	147 (15)
Race	
Caucasian, n (%)	686 (72)
Black, n (%)	166 (17)
Asian, n (%)	2 (0.2)
Other, n (%)	102 (10.8)
Ethnicity	
Hispanic/Latino, n (%)	162 (17)
Not Hispanic/Latino, n (%)	794 (83)
BMI, median (range)	27.9 (14.3, 66.5)
Weight, median (range), kilograms	85 (43.9, 185.2)
CD4^+^ cell count, median (range), cells/mm^3^	699 (43, 2504)
Documented duration of HIV infection prior to switch, median (range), years	15 (2, 38)
Documented duration of ART prior to switch, median (range), years	11 (1, 37)
Documented duration of virologic suppression prior to switch, median (range), years	9 (1, 31)
BIC/F/TAF switches	673
*Regimen prior to switch*	
Dual NRTI + NNRTI, n (%)	148 (22)
Dual NRTI + PI, n (n%)	69 (10)
Dual NRTI + INSTI, n (%)	401 (60)
Other, n (n%)	55 (8)
*NRTI prior to switch*	
TAF, n (%)	472 (70)
TDF, n (%)	135 (20)
ABC, n (%)	19 (3)
*Anchor drug prior to switch*	
DTG, n (%)	103 (16)
RAL or EVG/c, n (%)	298 (44)
DRV or ATV, n (%)	70 (10)
EFV, n (%)	89 (13)
RPV, n (%)	37 (5)
*Rationale for switch to BIC/F/TAF*	
Simplification, n (%)	197 (29)
DDI avoidance, n (%)	134 (20)
TDF to TAF switch	1 (0.15)
Comorbidities, n (%)	17 (3)
Side effects, n (%)	95 (14)
Other, n (%)	229 (33.85)
DBR switches	283
DTG + F/TAF	148 (52)
DTG/ABC/3TC	51 (18)
DTG/RPV	48 (17)
DTG/3TC	36 (13)
*Regimen prior to switch*	
Dual NRTI + NNRTI, n (%)	43 (15)
Dual NRTI + PI, n (n%)	42 (15)
Dual NRTI + INSTI, n (%)	147 (52)
Other, n (n%)	51 (18)
*NRTI prior to switch*	
TAF, n (%)	64 (23)
TDF, n (%)	116 (41)
ABC, n (%)	45 (16)
*Anchor drug prior to switch*	
DTG, n (%)	91 (32)
RAL or EVG/c, n (%)	50 (18)
DRV or ATV, n (%)	39 (14)
EFV, n (%)	17 (6)
RPV, n (%)	21 (7)
*Rationale for switch to DBR*	
Simplification, n (%)	105 (37)
DDI avoidance, n (%)	25 (9)
TDF to TAF switch	38 (13)
Comorbidities, n (%)	14 (5)
Side effects, n (%)	44 (16)
Other, n (%)	57 (20)

ABC = abacavir, ART = antiretroviral therapy, ATV = atazanavir, BIC/F/TAF = bictegravir/emtricitabine/tenofovir alafenamide, BMI = body mass index, c = cobicistat, DBR = dolutegravir-based regimen, DDI = drug–drug interaction, DRV = darunavir, DTG = dolutegravir, EFV = efavirenz, EVG = elvitegravir, F = emtricitabine, INSTI = integrase strand transfer inhibitor, NNRTI = non-nucleoside reverse transcriptase inhibitor, NRTI = nucleoside reverse transcriptase inhibitor, PI = protease inhibitor, RAL = raltegravir, RPV = rilpivirine, TAF = tenofovir alafenamide, 3TC = lamivudine, TDF = tenofovir disoproxil fumarate.

**Figure 1.**
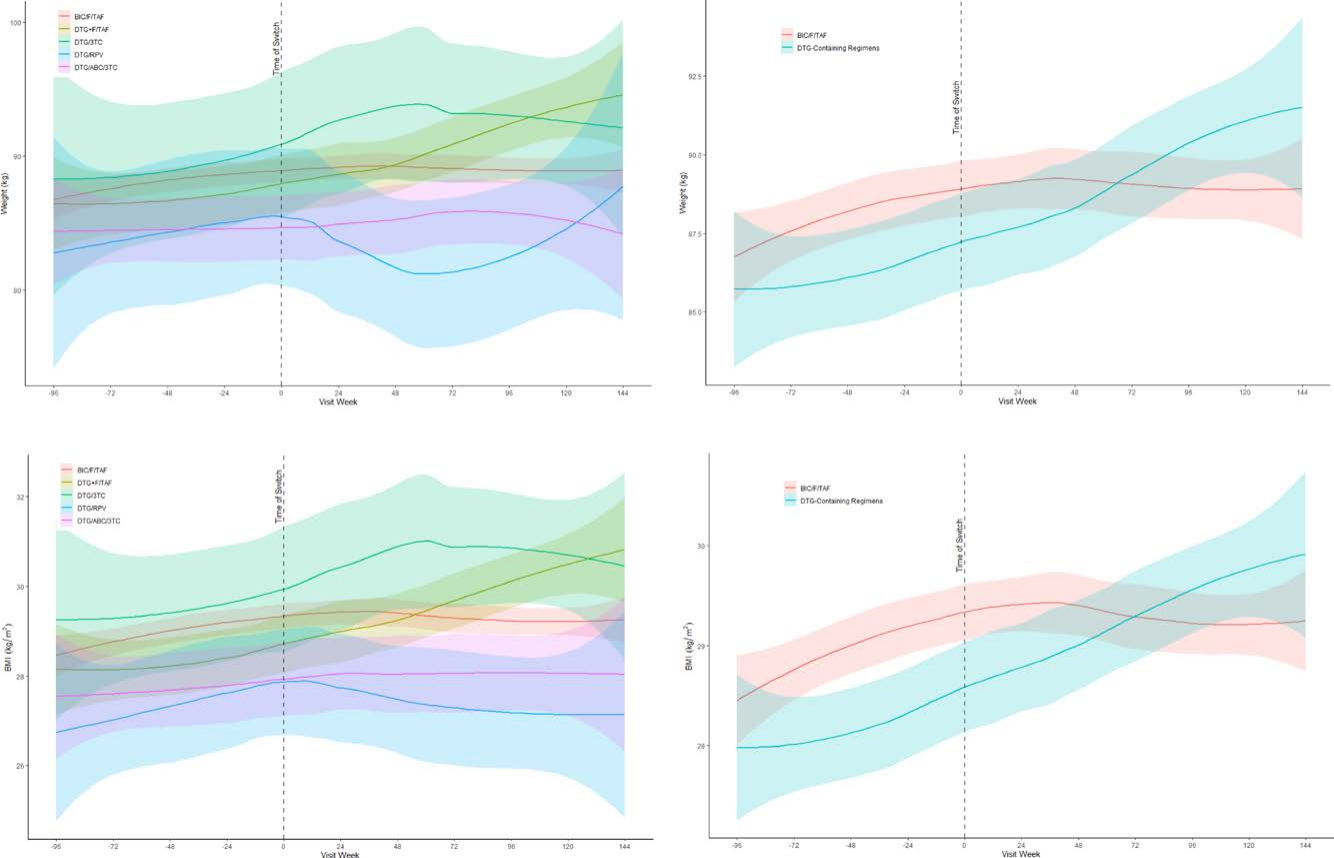
Mean change in weight and BMI before and after switch to B/F/TAF versus a dolutegravir-based regimen through week 144. B/F/TAF = bictegravir/emtricitabine/tenofovir alafenamide, BMI = body mass index.

At week 144, switching to B/F/TAF versus a DBR were both associated with lower annualized weight gain post-switch compared to pre-switch (−0.88 kg/year vs −0.39 kg/year respectively, *P* = .15). Similar trends were observed for changes in BMI with reductions of −0.3 kg/m^2^/year and −0.12 kg/m^2^/year, *P* = .17 in the B/F/TAF and DBR groups respectively (Fig. 1). In adjusted models, PLWH in the B/F/TAF switch group had an annualized weight gain of +1.13 kg/year pre-switch compared to a decreased annualized weight gain of +0.38 kg/year post-switch with a pre-and-post-switch difference of −0.75 kg/year which was found to be statistically significant (*P ≤* .001). PLWH in the DBR switch group had an annualized weight gain of + 1.17 kg/year pre-switch compared to a decreased annualized weight gain of + 0.78 kg/year post-switch with a pre-and-post-switch difference of −0.39 kg/year which was not found to be statistically significant (*P* = .16). Similar trends were observed for BMI changes (Fig. 1). PLWH switched to B/F/TAF had lower annualized weight gain post-switch compared to all DBRs except for those switched to DTG/RPV (Fig. 1, Table 2). Among those switched to DBRs, the highest annualized weight gain post-switch was observed among those switched to DTG plus F/TAF (+0.68 kg/year, 95% CI: −0.32, 1.65) whereas the lowest annualized weight gain post-switch was observed among those switched to DTG/RPV (−2.22 kg/year, 95% CI: −3.69, −0.62) (Table 2). PLWH switching to DTG/RPV and B/F/TAF were the only groups with significantly lower annualized weight gain post-switch, no treatment group was associated with significantly higher annualized weight gain post-switch (Table 2).

**Table 2:** Adjusted annualized mean weight change pre-/-post switch to B/F/TAF versus a dolutegravir-based regimen through 144 weeks.

	All N = 956	B/F/TAF N = 673	DBRs (grouped) N = 283	DTG + F/TAF N = 148	DTG/ABC/3TC N = 51	DTG/RPV N = 48	DTG/3TC N = 36
Pre-switch kg/year (95% CI)	1.13 (0.87, 1.39)*	1.12 (0.81, 1.43)*	1.17 (0.68, 1.66)*	1.1 (0.22, 1.98)*	0.7 (-0.01, 1.41)	1.05 (-0.47, 2.55)	1.35 (0.38, 2.31)*
Post-switch kg/year (95% CI)	0.38 (0.14, 0.61)*	0.23 (-0.04, 0.51)	0.78 (0.34, 1.22)*	1.78 (0.97, 2.56)*	0.32 (-0.33, 0.98)	-1.16 (-2.53, 0.27)	1.08 (0.31, 1.85)*
Pre-post difference kg/year (95% CI)	-0.75 (-1.05, −0.45)	-0.88 (-1.24, −0.53)*	-0.39 (-0.92, 0.15)	0.68 (-0.32, 1.65)	-0.38 (-1.16, 0.41)	-2.22 (-3.69, −0.62)*	-0.27 (-1.32, 0.79)

ABC = abacavir, B/F/TAF = bictegravir/emtricitabine/tenofovir alafenamide, CI = confidence interval, DBR = dolutegravir-based regimen, DTG = dolutegravir, RPV = rilpivirine, 3TC = lamiuvidine.

* Significant *P*-value <.05.

Through week 144, there were significantly more cases of incident hypertension observed among those on DTG/3TC (4/17, 24%) and DTG/ABC/3TC (6/37, 16%) (Table 3). There were also significantly more incident cases of MASLD in the DTG/3TC group (4/34, 12%), whereas more incident cases of hyperlipidemia were observed among those on DTG/RPV (3/9, 33%). There was a trend towards more PLWH on DTG plus F/TAF (15/136, 11%), DTG/RPV (6/48, 13%) and DTG/3TC (4/30, 13%) starting new medications for diabetes mellitus and more PLWH on B/F/TAF (45/667, 7%) and DTG/3TC (3/35, 9%) being referred to the OIC wellness clinic though these were not statistically significant. There were no other significant differences in the proportions newly diagnosed with cardiometabolic disease, initiating pharmacotherapy for cardiometabolic conditions, referred to the OIC wellness clinic or starting weight loss pharmacotherapy (Table 3). Notably, lipid panels and HbA1c values were available for <7% of the overall cohort therefore changes in these parameters by treatment group were unable to be analyzed.

**Table 3:** Change in cardiometabolic factors among adults switching to B/F/TAF versus a dolutegravir-based regimen from baseline to week 144.

	B/F/TAF N (%)	DTG + F/TAF N (%)	DTG/ABC/3TC N (%)	DTG/RPV N (%)	DTG/3TC N (%)	*P*-value
New HTN diagnosis	20/387 (5)	11/93 (12)	6/37 (16)	0/29 (0)	4/17 (24)	**.001**
New DM2 diagnosis	19/579 (3)	8/127 (6)	2/46 (4)	0/39 (0)	2/28 (7)	.21
New obesity diagnosis	21/517 (4)	9/120 (8)	1/42 (2)	3/39 (8)	1/25 (4)	.36
New MASLD diagnosis	8/637 (1)	0/137 (0)	2/51 (4)	2/47 (4)	4/34 (12)	**.001**
New HLD diagnosis	16/252 (6)	9/75 (12)	2/24 (8)	3/39 (33)	1/10 (10)	**.044**
Started HTN medications	66/386 (17)	14/89 (16)	9/50 (18)	6/48 (13)	6/16 (38)	.28
Discontinued HTN medications	48/673 (7)	14/147 (10)	4/51 (8)	3/48 (6)	6/36 (17)	.28
Started DM2 medications	35/590 (6)	15/136 (11)	2/51 (4)	6/48 (13)	4/30 (13)	.05
Discontinued DM2 medications	18/672 (3)	3/148 (2)	2/51 (4)	1/48 (2)	2/36 (6)	.62
Started Vit E for MASLD	10/659 (2)	0/145 (0)	1/51 (2)	0/48 (0)	1/32 (3)	.28
Discontinued Vit E for MASLD	3/671 (0.4)	0/148 (0)	0/51 (0)	0/48 (0)	0/36 (0)	.99
Started HLD medications	65/370 (18)	17/95 (18)	10/50 (20)	11/47 (23)	3/18 (17)	.87
Discontinued HLD medications	68/670 (10)	18/148 (12)	2/51 (4)	7/48 (15)	2/36 (6)	.33
Referral to OIC Wellness clinic	45/667 (7)	4/148 (3)	0/51 (0)	1/48 (2)	3/35 (9)	.05
Started weight loss medications	65/590 (11)	15/133 (11)	2/46 (4)	3/44 (7)	6/28 (21)	.22

Significant *P*-values have been bolded for ease of interpretation.

Variability in denominator due to missing data.

ABC = abacavir, B/F/TAF = bictegravir/emtricitabine/tenofovir alafenamide, DM2 = type 2 diabetes, DTG = dolutegravir, HLD = hyperlipidemia, HTN = hypertension, MASLD = metabolic dysfunction-associated steatotic liver disease, OIC = Orlando Immunology Center, RPV = rilpivirine, 3TC = lamivudine, Vit E = vitamin E.

Baseline BMI < 18.5 kg/m^2^ was the only evaluated factor associated with significantly higher annualized weight gain post-switch, whereas multiple factors were associated with significantly lower annualized weight gain post-switch but among them self-report of dieting (−2.67 kg/year, 95% CI: −3.96, −1.37), switching from a PI (−2.26 kg/year, 95% CI: −3.27, −1.23), baseline BMI > 30 kg/m^2^ (−2.24 kg/year, 95% CI: −2.88, −1.61) and self-report of physical activity (−2.23 kg/year, 95% CI: −3.28, −1.17) were associated with the lowest annualized weight gain post-switch (Table 4).

**Table 4:** Factors associated with annualized weight change following switch to BIC/F/TAF versus a dolutegravir-based regimen.

Characteristic	Pre-switch kg/year 95% CI	Post-switch kg/year 95% CI	Pre-post difference kg/year 95% CI
Age			
<50 years	1.46 (1.03, 1.89)*	0.42 (0.04, 0.80)*	-1.04 (-1.54, -0.54)*
≥50 years	0.85 (0.53, 1.18)*	0.35 (0.06, 0.64)*	-0.51 (-0.87, -0.15)*
Sex			
Male	1.15 (0.87, 1.42)*	0.48 (0.25, 0.72)*	-0.66 (-0.98, -0.34)*
Female	1.07 (0.25, 1.89)*	-0.27 (-1.07, 0.52)	-1.34 (-2.14, -0.55)*
Race/ethnicity			
Caucasian	1.08 (0.72, 1.44)*	0.38 (0.08, 0.69)*	-0.7 (-1.11, -0.28)*
Black	1.4 (0.75, 2.06)*	0.15 (-0.47, 0.77)	-1.25 (-1.92, -0.59)*
Hispanic/Latino	0.96 (0.51, 1.42)*	0.63 (0.22, 1.04)*	-0.33 (-0.86, 0.2)
Other	1.18 (-0.58, 2.96)	0.54 (-1.12, 2.26)	-0.64 (-1.97, 0.65)
Baseline BMI			
<18.5 kg/m^2^	-0.74 (-1.92, 0.46)	1.38 (0.2, 2.57)*	2.13 (0.26, 3.88)*
18.5–24.9 kg/m^2^	0.52 (0.17, 0.87)*	0.79 (0.48, 1.09)*	0.27 (-0.16, 0.69)
25–29.9 kg/m^2^	0.75 (0.38, 1.12)*	0.65 (0.32, 0.98)*	-0.1 (-0.52, 0.31)
≥30 kg/m^2^	2.02 (1.45, 2.6)*	-0.22 (-0.74, 0.29)	-2.24 (-2.88, -1.61)*
Nadir CD4^+^ T-cell count			
<200 cells/mm^3^	1.37 (0.88, 1.87)*	0.76 (0.33, 1.2)*	-0.61 (-1.19, -0.03)*
≥200 cells/mm^3^	0.99 (0.69, 1.3)*	0.15 (-0.12, 0.42)	-0.84 (-1.18, -0.51)*
Baseline CD4^+^ T-cell count			
<200 cells/mm^3^	2.64 (0.89, 4.39)*	0.49 (-1.2, 2.17)	-2.15 (-4.29, 0.01)*
≥200 cells/mm^3^	1.09 (0.83, 1.36)*	0.37 (0.14, 0.61)*	-0.72 (-1.02, -0.42)*
Duration of HIV infection			
0–5 years	2.07 (1.28, 2.86)*	0.6 (-0.12, 1.3)	-1.47 (-2.33, -0.62)*
6–10 years	1.45 (0.95, 1.95)*	0.4 (-0.03, 0.82)	-1.05 (-1.67, -0.44)*
>10 years	0.78 (0.45, 1.12)*	0.31 (0.01, 0.61)*	-0.47 (-0.84, -0.1)*
Pre-switch anchor drug			
NNRTI	1.00 (0.53, 1.47)*	1.00 (0.58, 1.42)*	0.00 (-0.57, 0.56)
PI	1.89 (0.98, 2.8)*	-0.37 (-1.19, 0.45)	-2.26 (-3.27, -1.23)
INSTI	1.02 (0.69, 1.35)*	0.32 (0.03, 0.61)*	-0.7 (-1.06, -0.34)*
Pre-switch NRTI			
TAF	1.25 (0.91, 1.6)*	0.05 (-0.26, 0.35)	-1.21 (-1.61, -0.81)*
TDF	1.08 (0.61, 1.55)*	1.13 (0.71, 1.55)*	0.05 (-0.48, 0.57)
ABC	0.47 (-0.24, 1.18)	0.51 (-0.11, 1.15)*	0.04 (-0.67, 0.76)
Baseline smoker			
Yes	1.44 (0.7, 2.19)*	0.45 (-0.23, 1.14)	-0.99 (-1.77, -0.21)*
No	1.06 (0.78, 1.34)*	0.37 (0.12, 0.61)*	-0.7 (-1.02, -0.37)*
Baseline psychiatric comorbidities			
Yes	1.09 (0.66, 1.52)*	0.26 (-0.13, 0.64)	-0.84 (-1.31, -0.36)*
No	1.16 (0.82, 1.49)*	0.46 (0.17, 0.76)*	-0.70 (-1.08, -0.32)*
Use of medications associated with weight gain			
Yes	0.92 (0.41, 1.43)*	0.01 (-0.44, 0.47)	-0.91 (-1.47, -0.34)*
No	1.24 (0.94, 1.54)*	0.58 (0.31, 0.84)*	-0.66 (-1.01, -0.32)*
Use of medications associated with weight loss			
Yes	1.18 (0.56, 1.8)*	-0.03 (-0.56, 0.5)	-1.21 (-1.89, -0.54)*
No	1.11 (0.82, 1.4)*	0.5 (0.24, 0.76)*	-0.61 (-1.01, -0.32)*
Referral to OIC wellness clinic			
Yes	0.63 (-0.59, 1.85)	0.12 (-0.9, 1.11)	-0.52 (-1.94, 0.92)
No	1.16 (0.89, 1.43)*	0.4 (0.16, 0.64)*	-0.76 (-1.07, -0.46)*
Self-report of physical activity			
Yes	1.47 (0.52, 2.43)*	-0.75 (-1.61, 0.1)	-2.23 (-3.28, -1.17)*
No	1.06 (0.79, 1.32)*	0.46 (0.23, 0.7)*	-0.59 (-0.89, -0.3)*
Self-report of dieting			
Yes	1.44 (0.24, 2.64)*	-1.23 (-2.29, -0.17)*	-2.67 (-3.96, -1.37)*
No	1.05 (0.78, 1.31)*	0.47 (0.24, 0.71)*	-0.57 (-0.87, -0.28)*

ABC = abacavir, BMI = body mass index, CI = confidence interval, INSTI = integrase strand transfer inhibitor, NNRTI = non-nucleoside reverse transcriptase inhibitor, NRTI = nucleoside reverse transcriptase inhibitor, OIC = Orlando Immunology Center, PI = protease inhibitor, TAF = tenofovir alafenamide, TDF = tenofovir disoproxil fumarate.

* Significant *P*-value <.05.

## 4. Conclusions

In this real-world cohort, switching to a BIC versus DBR were both associated with lower annualized weight gain post-switch that was not significantly different at week 144 with similar changes in BMI noted. These findings are consistent with prior studies which have demonstrated no significant changes in weight and BMI following switch to a second-generation INSTI however few included PLWH switched to B/F/TAF.^[13,18–20]^ In pooled data from RCTs, switching to BIC and DTG were not associated with an increased risk of ≥ 10% weight gain at week 48 and weight change was not significantly different among those switching from DTG to BIC versus those remaining on DTG.^[13]^ Our results from a large diverse cohort switched to B/F/TAF versus a DBR overall support these findings and also demonstrated no significant weight changes among those switched to BIC versus DTG. In contrast, other studies have reported increased weight following switch to second-generation INSTIs, including the PASO-DOBLE RCT which revealed significantly more weight gain among those switching to BIC versus DTG.^[3,8–10,14]^ However, many of these analyses evaluated a significant proportion switched from TDF and EFV, 2 agents thought to be weight-suppressive whose removal from a regimen generally leads to weight gain.^[15,16]^ Our cohort only included 26% switching from TDF and 11% switching from EFV hence our findings further support the weight neutrality of second-generation INSTIs which were not associated with excess weight gain in a population mostly switching from other regimens considered to be more weight neutral. Notably, there were more switching from TDF to a DBR compared to B/F/TAF (41% vs 20% respectively), however use of TDF pre-switch was not associated with significantly more weight gain post-switch in adjusted models.

At week 144, the only treatment groups with significant weight changes post-switch were those switched to DTG/RPV and B/F/TAF, both groups had significantly lower weight gain post-switch (−2.22 kg/year (95% CI: −3.69, −0.62) and −0.88 kg/year (95% CI: −1.24, −0.53) respectively). To date this is one of few studies reporting weight changes in people switched to DTG/RPV. Data from a sub analysis of the DOLBi study demonstrated a small but significant weight gain of 2.5% in the first year following switch to DTG/RPV among 37 stably suppressed PLWH.^[25]^ This was accompanied by fat mass gain without changes in lean mass or obesity prevalence. Predictors of fat mass increase in this cohort included lower baseline fat mass and CD4^+^ T-cell count counts. The median baseline BMI and CD4 + T-cell count among PLWH switching to DTG/RPV in our cohort were 26.78 kg/m^2^ and 652 cells/mm^3^ respectively hence differences in baseline characteristics of the cohorts may account for these opposing observations. A closer look at reasons for switch in our DTG/RPV arm also revealed that 12/48 (25%) switched “to improve metabolic comorbidities” hence switching to DTG/RPV in these individuals may have been 1 component of an effort to improve metabolic health. We hypothesize that they may have also received more encouragement to make healthy lifestyle choices at the time of switch and this may explain why DTG/RPV switches lost more weight post-switch compared to other treatment groups.

B/F/TAF switches also lost significantly more weight post-switch and several RCTs evaluating weight change following switch to BIC from PIs and other INSTI-based regimens have not demonstrated any significant weight changes post-switch.^[26,27]^ Observational data from 236 PLWH switching from a non-INSTI to an INSTI-based regimen also reported weight gain with switch to all INSTIs except BIC.^[9]^ Our findings however differ from other RCTs and observational studies which revealed increased weight following switch to BIC^[10,14]^ however as previously noted many of these studies included a significant proportion switching from TDF and EFV which may explain our differing results.

There were small but significant differences in incident hypertension, MASLD and hyperlipidemia at week 144, with many of these new diagnoses occurring among those on DBRs. Other studies have also reported worsening cardiometabolic outcomes with the use of INSTIs, particularly DTG^[21–23]^ whereas some cohorts have reported no significant change in metabolic parameters or incident cardiometabolic disease following INSTI initiation.^[13,24]^ Our results add to this pool of data with evaluation of a comprehensive and clinically relevant group of cardiometabolic parameters with no significant differences observed by treatment arm in many of the factors studied. Notably, the sample size in many of these groups was small due to missing data in the EMR and these findings should be interpreted with caution.

In our cohort, baseline BMI < 18.5 kg/m^2^ was the only evaluated factor associated with significantly higher weight gain post-switch at week 144. This is consistent with data from multiple studies which have demonstrated that being underweight at ART initiation or switch is a risk factor for excess weight gain and in fact may reflect a “return to health.”^[1,4,5,15]^ In the case of switches to a second-generation INSTI such as BIC or DTG, the greater weight gain post-switch in those with low baseline BMI may also reflect better gastrointestinal tolerability of these agents thereby enabling improved appetite and fewer weight attenuating side effects such as nausea and diarrhea. At week 144, multiple factors were associated with lower annualized weight gain post-switch, but among these self-report of dieting was associated with the lowest weight gain post-switch. Calorie restriction has long been identified as an effective means of weight loss in the general population^[28,29]^ and several studies have proven its benefit in reducing obesity and cardiometabolic complications in PLWH.^[30–32]^ Ideally, it is usually recommended in combination with exercise and self-report of physical activity was also associated with significantly lower annualized weight gain post-switch in this study. Together this suggests that PLWH suffering from obesity and its related complications may benefit from programs providing education on healthy nutrient intake, and exercise to optimize their cardiometabolic health.

Limitations of this study include the observational nature of the analysis which limits our ability to control for other confounding factors that impact weight and cardiometabolic disease. Our study did not include a control “non-switch” group; however, we did utilize a design comparing pre-and post-switch weight and BMI changes which allowed participants to serve as their own control reducing confounding factors such as diet, exercise and stress levels which may affect weight. Additional limitations are that data are from a single center in the Southeastern United States, there were small sample sizes in many of the DBR groups and the study population only comprised 15% of cisgender women which warrants caution with generalizability and interpretation of these results. We were unable to include data on detailed body fat analyses such as those obtained from dual-energy x-ray absorptiometry scans and body circumference measurements as these are not routinely collected in clinical practice. Hence it is unknown whether the weight loss observed in our study corresponds to fat versus lean muscle mass and which areas of the body were most affected. Future analyses examining weight and BMI changes among PLWH should aim to include assessments of dual-energy x-ray absorptiometry-quantified body composition to determine if weight changes correlate with changes in body fat or fat-free “muscle” mass as the former may be undesired and not associated with any metabolic benefit.

The analysis of cardiometabolic factors was also limited by missing data in the EMR which led to an inability to compare changes in lipid parameters and HbA1c values by treatment group as very few participants in the overall cohort had these data available for evaluation. Missing data also resulted in smaller sample sizes in certain treatment groups, that is, DTG/3TC potentially impacting the significance of these findings. We also note that our assessments did not include endpoints related to changes in the dose of medications used to treat cardiometabolic disease but rather was limited to whether these drugs were newly prescribed or discontinued during the study period. However, this is one of few studies providing long-term data on changes in weight, BMI and incident cardiometabolic disease among PLWH switching to BIC versus DTG and these findings provide additional insight on whether a differential impact on these metabolic parameters exists among those treated with these regimens. Additional studies are needed to determine how changes in weight following ARV switch correlate with changes in new-onset cardiometabolic disease and the use of medications to treat the latter.

In summary, we observed lower annualized weight gain following switch to B/F/TAF and DBRs among a large diverse cohort through 144 weeks with no significant differences in weight change between B/F/TAF and DBRs. This study further supports the weight neutrality of BIC and DTG-based regimens and provides further evidence that they have few significant differences in new-onset cardiometabolic disease which is important given the aging population living with HIV and the increasing number of medical comorbidities they face. For PLWH struggling with weight gain, referral to services that provide counseling on nutrition, exercise and pharmacotherapy for weight loss may be a valuable intervention.

## Author contributions

**Conceptualization:** Charlotte-Paige Rolle, Jeffrey Garrett, Kiran Patel, Federico Hinestrosa, Edwin DeJesus.

**Data curation:** Charlotte-Paige Rolle, Jamie Castano.

**Formal analysis:** Charlotte-Paige Rolle, Vu Nguyen.

**Funding acquisition:** Charlotte-Paige Rolle.

**Investigation:** Charlotte-Paige Rolle.

**Methodology:** Charlotte-Paige Rolle, Vu Nguyen.

**Project administration:** Charlotte-Paige Rolle.

**Resources:** Charlotte-Paige Rolle.

**Software:** Charlotte-Paige Rolle.

**Supervision:** Charlotte-Paige Rolle, Edwin DeJesus.

**Validation:** Charlotte-Paige Rolle.

**Visualization:** Charlotte-Paige Rolle.

**Writing – original draft:** Charlotte-Paige Rolle, Jeffrey Garrett, Vu Nguyen.

**Writing – review & editing:** Charlotte-Paige Rolle, Jeffrey Garrett, Vu Nguyen, Kiran Patel, Federico Hinestrosa, Edwin DeJesus.
